# PIEZO2 expression is an independent biomarker prognostic for gastric cancer and represents a potential therapeutic target

**DOI:** 10.1038/s41598-023-48577-5

**Published:** 2024-01-12

**Authors:** Yun-Chao Zhang, Min Yang, Cen-di Lu, Quan-Yao Li, Jin-na Shi, Jun Shi

**Affiliations:** 1 Department of Oncology, Yueyang Hospital of Integrated Traditional Chinese and Western Medicine, Shanghai University of Traditional Chinese Medicine, Shanghai, 200437 People’s Republic of China; 2 Department of Oncology, The Second Affiliated Hospital of Zhejiang Chinese Medical University, 318 Chaowang Road, Hangzhou, 310000 Zhejiang People’s Republic of China; 3 Department of Traditional Chinese Medicine, Shanghai Fourth People’s Hospital Affiliated to Tongji University School of Medicine, Shanghai, 200434 People’s Republic of China

**Keywords:** Cancer genomics, Gastrointestinal cancer, Oncogenes, Tumour biomarkers, Tumour immunology

## Abstract

Gastric cancer (GC) is one of the most prevalent malignant tumors of the gastrointestinal system in the globe. The effect of PIEZO2 on the immune function and pathological features of gastric cancer remains to be explored. The Online database of cancer genes and GSE54129 have been used to analyze the clinical characteristics of PIEZO2 expression. We looked at the relationship between PIEZO2 and the immune systems of GC patients. The TIDE algorithm was used to explore the value of PIEZO2 in immunotherapy. Investigated the enrichment of PIEZO2 gene ontology and associated signal pathways using Online gene databases. The results show that overexpression of PIEZO2 was identified as an independent risk factor for patients with GC who had poor overall survival. Individuals may have a better prognosis if they had poorly differentiated GC and increased PIEZO2 expression (*P* < 0.05). We demonstrated a strong correlation between PIEZO2 and immune cells. The majority of immune checkpoint and immunological-related genes were associated with PIEZO2 expression. And PIEZO2 might be used as an immunotherapy target. Finally, the differential PIEZO2 genes in GC were mostly implicated in the processes of inflammation, immunological response, and tumor metastasis, according to functional analysis. PIEZO2 has a negative correlation with cell stemness and mutation levels in patients with GC and a positive correlation with immune cell infiltration and gene expression in the tumor microenvironment. These findings point to PIEZO2 as a potential new immunotherapy target of GC.

## Introduction

Gastric cancer (GC) is one of the most prevalent digestive malignancies worldwide. A recent study shows that globally its incidence rate ranks fifth and its mortality ranks fourth^1^. Current treatment methods for GC include endoscopic examination, gastrectomy, and chemotherapy or adjuvant chemotherapy or neoadjuvant therapy. Although treatment efficacy has improved, outcomes are still very poor^2^. Finding possible biomarkers for early diagnosis or therapy targets to enhance patient outcomes for GC is therefore extremely important.

The transmembrane protein PIEZO2 is a member of the PIEZO family and is crucial for the quick response of somatosensory neurons to mechanically generated currents. Its mechanical activation causes cations to flow into and activate additional Ca^2+^ channels, thus mediating Ca^2+^ influx, and regulating the cytoskeleton through RhoA activity^3^. Research shows that abnormal expression of PIEZO2 may be related to the prevalence and progression of cancer^3–5^. Although PIEZO2 has been found linked to prognostic in patients with GC^6, 7^. However, there is a lack of further exploration of the value of immunotherapy, potential mechanisms of immune function, and prognostic subgroup analysis in the GC.

This study uses the clinical data in the online database to conduct bioinformatics analysis on PIEZO2. We aimed to investigate the correlation of PIEZO2 with immune cell infiltration, immune-related genes, and immune checkpoint genes. Predicted the performance of PIEZO2 in immune checkpoint blocking therapy. The relationship between PIEZO2 and cancer stem cells and gene mutation in GC patients was analyzed, which saves time and work for clinical research in GC, and provides new therapeutic targets and research directions.

## Methods

### Analysis of PIEZO2 differential expression in the TCGA and GEO databases

High throughput sequencing data from the TCGA database (https://www.cancer.gov/tcga/), The expression matrix of the GSE54129 microarray was transformed into log2 in R after the original values of the data were retrieved from the GEO database. The University of California Santa Cruz (UCSC) Genome Browser Database (https://xenabrowser.net/) was used to retrieve the standardized pan-cancer dataset, and for each sample, the PIEZO2 gene expression data were extracted. The difference in PIEZO2 between tumor and normal tissues was assessed using the TCGA and GSE54129 datasets. We nalyse the ROC, obtained the AUC, and assessed the predictive value of PIEZO2 in tumor tissue and GC tissue using the R software package pROC (version 1.17.0.1).

GC tissue samples were separated using a median split into high and low-expression groups^8^. Then, using the t-test function of the R software program, the significance of each gene change between the comparison and control groups was assessed. The statistically significant screening threshold was set as absolute log2-fold change (FC) > 2 and adjusted *P* value < 0.01. A volcano map was used to visualize the up and down DEGs and display the DEGs related to PIEZO2 in a heat map. To nalyse the association between PIEZO2 and OS, first-progression survival, and post-progression survival, the query probe ID 1,562,488 was examined using the online tool Kaplan–Meier Plotter (https://kmplot.com/analysis/index.php?p=service&cancer=gastric) (PPS)^9^.

### Prediction of PIEZO2 and GC stemness and gene mutation

The TCGA Pan-Cancer dataset (PANCAN, N = 10,535, G = 60,499), which is unified and standardized, was acquired from the UCSC database. Each sample’s ENSG00000154864 (PIEZO2) gene expression information was taken from the database. Further, we screened the samples by methylation feature for each tumor obtained from previous research, EXPss, EREG-METHss, and RNAs tumor stemness scores^10^. The samples’ gene expression data and stemness index were combined. In addition, the MuTect2 program from GDC (https://portal.gdc.cancer.gov/) was used to process the basic nuclear variation data set of level 4 of all TCGA samples^11^. The samples’ gene expression data and stemness index were combined. In addition, the MuTect2 program from GDC (https://portal.gdc.cancer.gov/) was used to process the basic nuclear variation data set of level 4 of all TCGA samples^12^. The purity data of each tumor was obtained from a previous study^13^, and the TMB, MSI, purity, and gene expression data of the sample were integrated, and each expression value was further transformed using log2 (x + 0.001). After excluding cancer types with less than three samples of a single cancer type, the expression data of 37 cancer types were obtained.

### The development and evaluation of a prognostic model for GC patients

The data from survival time, survival status, and other pertinent features were combined using the R software package rms, and a nomogram was generated using the multifactor Cox technique to assess the predictive importance of these characteristics in 350 samples. The C index of the overall model is 0.66, 95% CI (0.61–0.71, p value = 1.4e−10).

### The relationship between PIEZO2 expression and immune cell infiltration and immune-related genes

Each expression value was then converted using log2 (x + 0.001) utilizing the UCSC database’s unified and standardized pan-cancer dataset. Furthermore, we took the gene expression profile for GC out of the database and mapped it to the GeneSymbol. The correlation of immune-related cells in patients with GC according to gene expression was reevaluated using the R software package IOBR and the deconvo XCell approach^14, 15^.

### Immune checkpoint analysis

We further retrieved the expression data of the PIEZO2 gene’s marker genes and a total of 60 genes from the immune checkpoint pathway (inhibitory (24), stimulatory (36)) from the standardized pan-cancer dataset from the UCSC database^13^. Additionally, all of the normal samples were filtered, and each expression value underwent a further log2(x + 0.001) transformation. The Pearson correlation between PIEZO2 and the marker genes for five immunological pathways was then calculated.

### Functional analyses of PIEZO2 in GC

We utilized the KEGG rest API (https://www.kegg.jp/kegg/rest/keggapi.html) to get the most recent gene annotation of the KEGG pathway for the functional enrichment analysis of the gene collection. The R software package org’s GO annotation of genes was utilized for the gene set function enrichment study. The background set utilized was Hs.eg.db (version 3.1.0). For GSEA, we used the GSEA software (version 3.0)^16^. We separated the samples into two groups based on PIEZO2 expression level (≥ 50%) and utilized c2.cp.kegg.v7.4.symbols.gmt subsets to nalyse important pathways and molecular processes^17^. We selected the lowest gene set as 5, the maximum gene set as 5000, and re-sampled 1000 times based on the gene expression profile and phenotypic categorization. A p-value of 0.05 and an FDR of 0.25 was regarded as statistically significant.

## Results

### The expression level of PIEZO2 in GC tissues is correlation with stemness and gene mutation 

**Figure 1:**
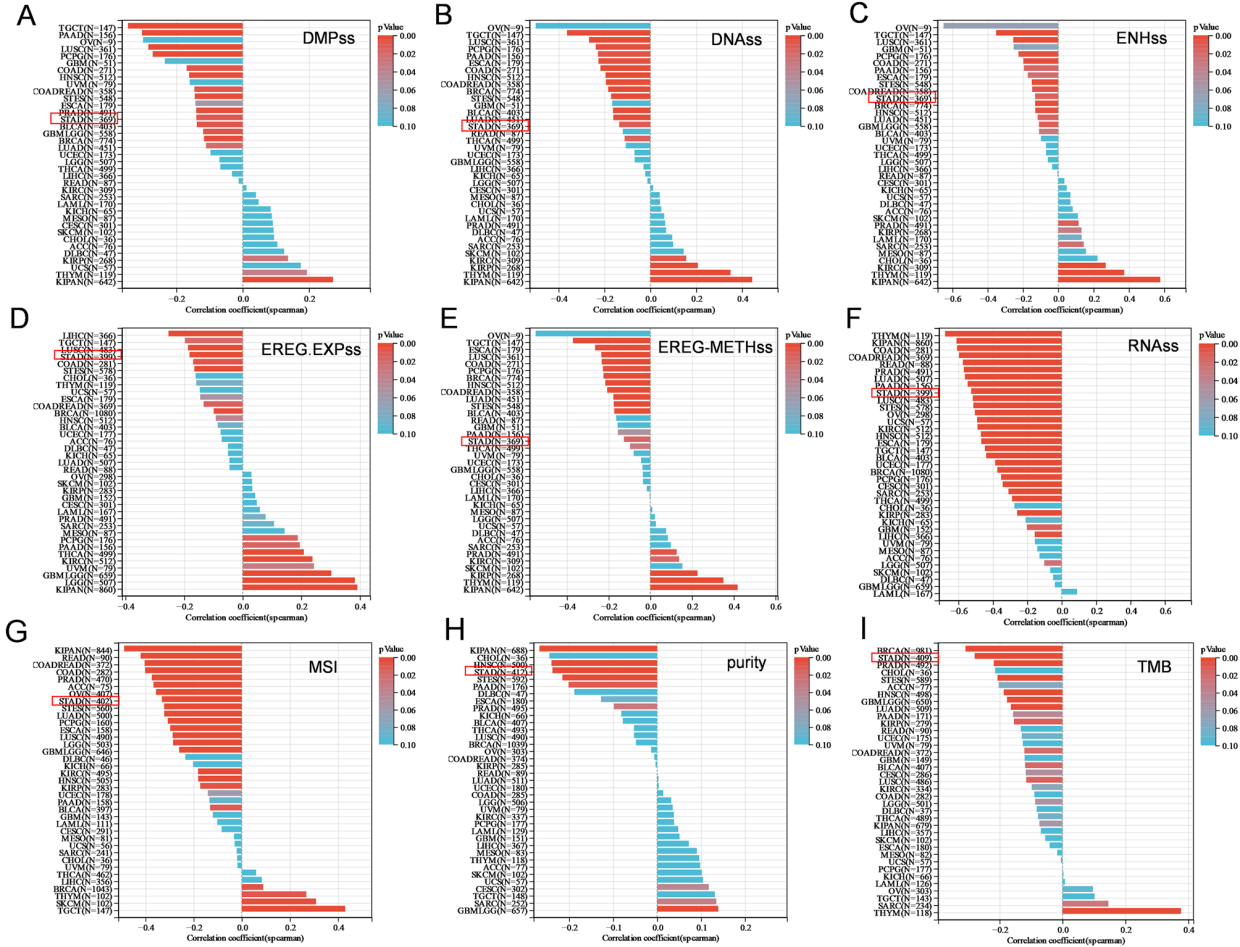
Pan-cancer analysis of PIEZO2 and tumor stemness related indicators (**A**–**I**) PIEZO2 negatively correlated with DMPss, DNAss, ENHss, EREG.EXPss, EREG-METHss and RNAss, microsatellite instability, purity, and tumor mutation burden in tumors of patients with gastric cancer (*P* < 0.05).

By analyzing the TCGA database and independent datasets, PIEZO2 can be used for the detection of GC tissue and is associated with late stages and worse prognosis of GC (Supplement Fig. S1, Supplement Table S1). And the expression of PIEZO2 is positively correlated with the gene expression of tumor progression and metastasis in GC (Supplement Fig. S2). Online Kaplan Meier Plotter analysis shows that PIEZO2 is not statistically correlated with female prognosis, but has an adverse association with male prognosis. However, in GC patients with poorly differentiated tumors, the PIEZO2 overexpression group had a better prognosis compared to other subgroups (Supplement Fig. S3, Supplement Table S2). According to research, poorly differentiated tumor cells have increased stemness, and tumor stem cells are frequently related to malignant development and poor prognosis of tumors. As a result, we examined the important PIEZO2 and tumor stemness markers in clinical patients. PIEZO2 was shown to be inversely linked with DMPss, DNAss, ENHss, and EREG.EXPss, EREG-METHss, RNAs, MSI, purity, and TMB in GC (Fig. 1A–J) (*P* < 0.05).

**Figure 2:**
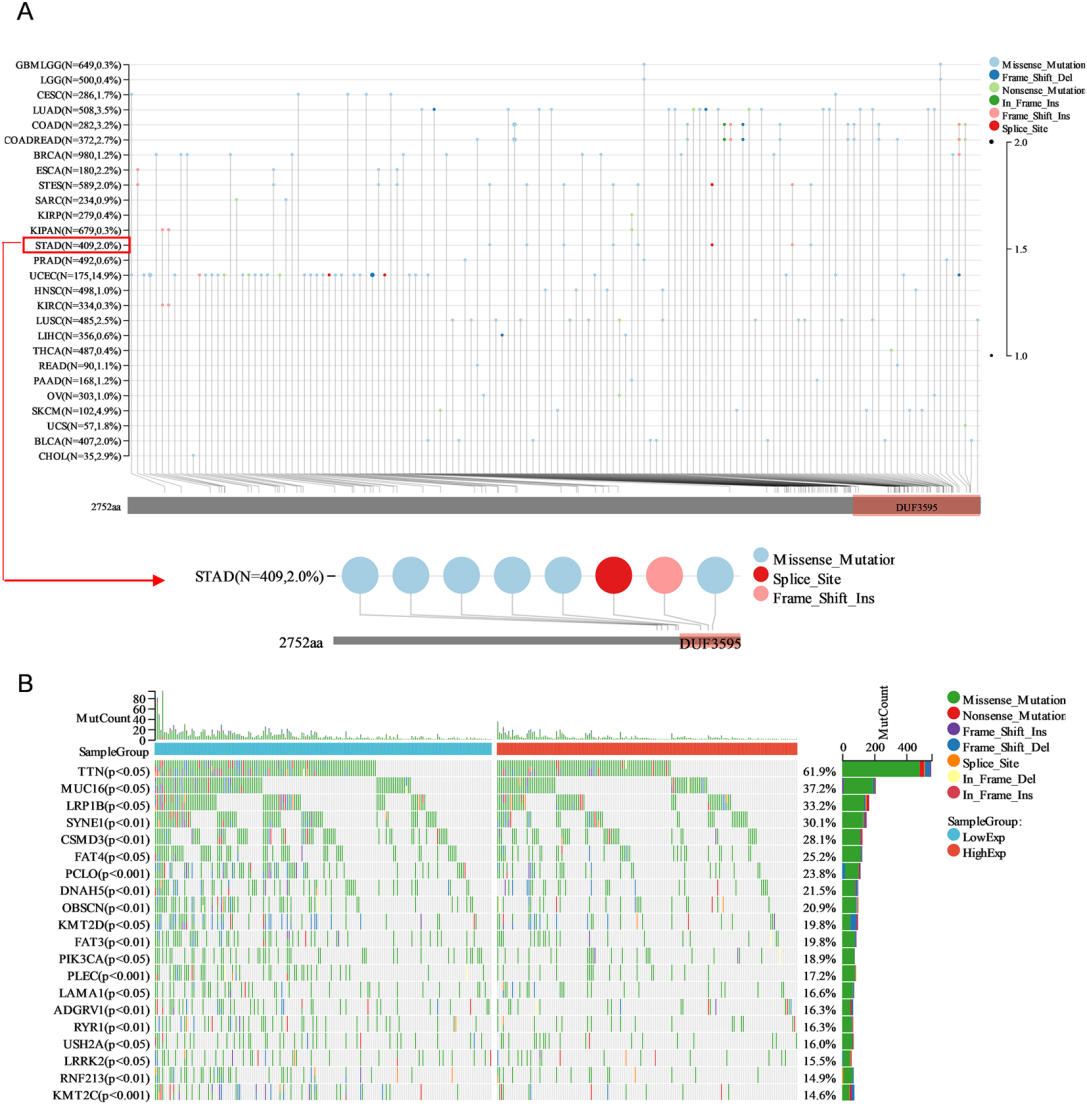
PIEZO2 mutation landscape (**A**) PIEZO2 mutation in gastric cancer. (**B**) Waterfall diagram showing the first 20 genes, mutation type, and number in the PIEZO2 high and low expression groups. *p < 0.05; **p < 0.01; ***p < 0.001; ****p < 0.0001; *ns* not significant.

This finding was supported further by an examination of the PIEZO2 mutation landscape. The findings revealed that the majority of PIEZO2 variants are missense, splice site, and frameshift insertion mutations (Fig. 2A). The waterfall graphic reveals that the PIEZO2 high expression group has much fewer mutations than the PIEZO2 low expression group (Fig. 2B). The findings imply that PIEZO2 may be adversely linked with tumor cell stemness and mutation in GC patients, resulting in a better prognosis in poorly differentiated tumors in GC patients with high PIEZO2 expression.

### Construction and computational validation of a nomogram model for patients with GC based on PIEZO2

**Figure 3:**
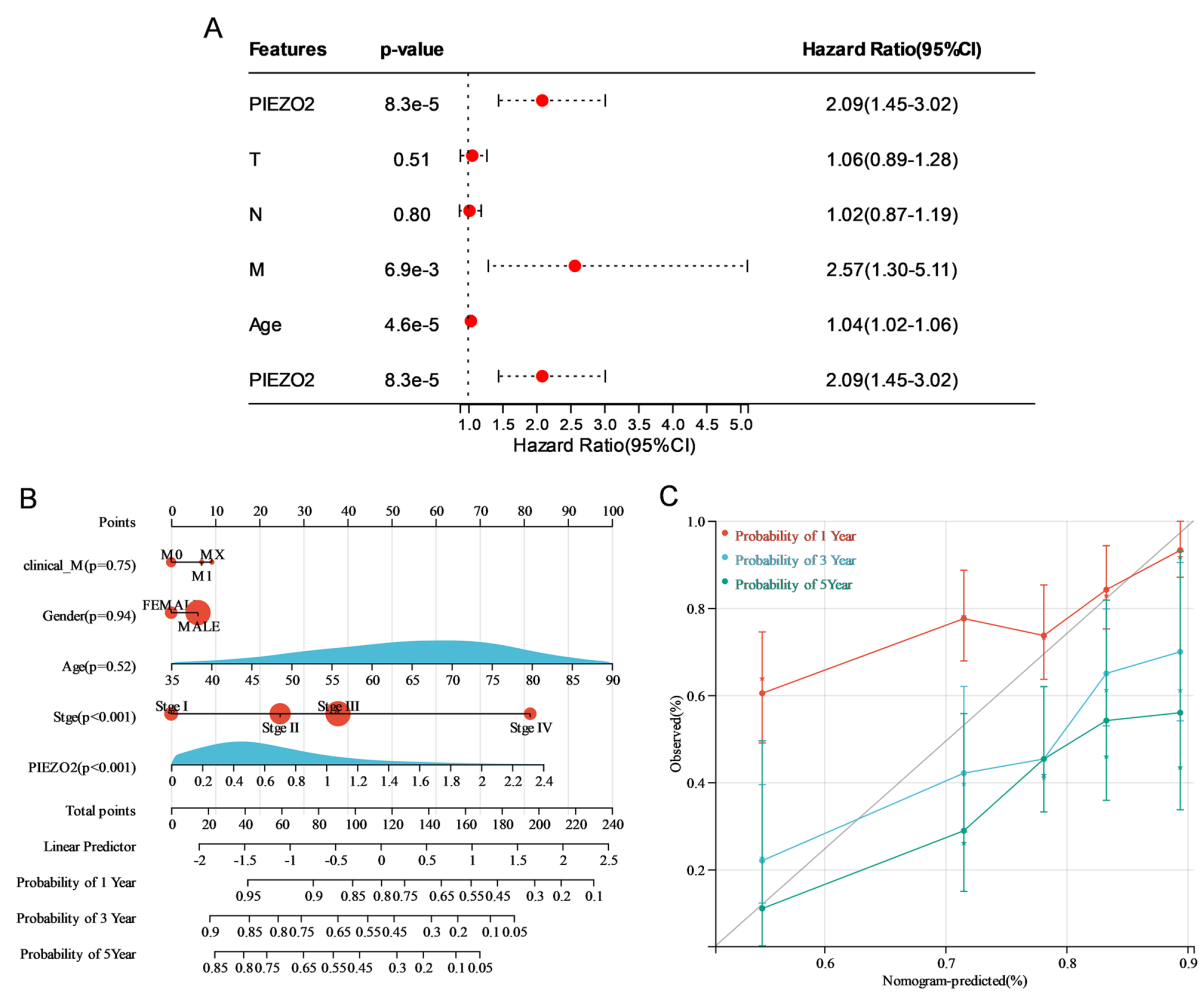
Prognostic factor analysis and nomogram model construction. (**A**) Forest chart showing the influence of multivariable Cox regression analysis-related factors on the overall survival rate of patients with gastric cancer (GC). (**B**) Nomogram model to predict the overall survival rate of patients with GC in 1, 3, and 5 years. (**C**) Calibration curve of nomogram model to predict the probability of 1, 3, and 5 year total survival of patients with GC. *p < 0.05; **p < 0.01; ***p < 0.001; ****p < 0.0001; *ns* not significant.

Multivariate Cox regression analysis showed that PIEZO2 (HR = 2.09, CI 1.45–3.02, *P* < 0.01), M stage (HR = 2.57, CI 1.30–5.11, *P* < 0.01), and age (HR = 1.04, CI 1.02–1.06, *P* < 0.01) were closely related to the prognosis of patients with GC (Fig. 3A). Based on these results, a nomogram model of OS (Fig. 3B) was constructed, and a score was assigned to each variable using the 100-score scale. The total prone score was calculated by adding the points for each variable, to give a score ranging from 0 to 240 points. Calibration curves drawn from the total subscale to the survival probability line were used to obtain the estimated probability of the total survival of patients with GC patients in 1, 3, and 5 years. The C index and calibration curve were used to assess the accuracy and reliability (Fig. 3C) of the prediction of the nomogram model. The C index of the nomogram model was 0.66, and 95% CI was 0.61–0.71, indicating that the model has some accuracy and might be used to predict the OS of GC patients. In the calibration plot, the bias-corrected lines of 1, 3, and 5 years were found to be near the ideal 45° diagonal, suggesting that the theoretical value was compatible with the observed value. The following findings demonstrate that the nomograph model may be used to forecast the overall survival rate of GC patients at 1, 3, and 5 years.

### The interaction of PIEZO2 with immune cells in GC patients

**Figure 4:**
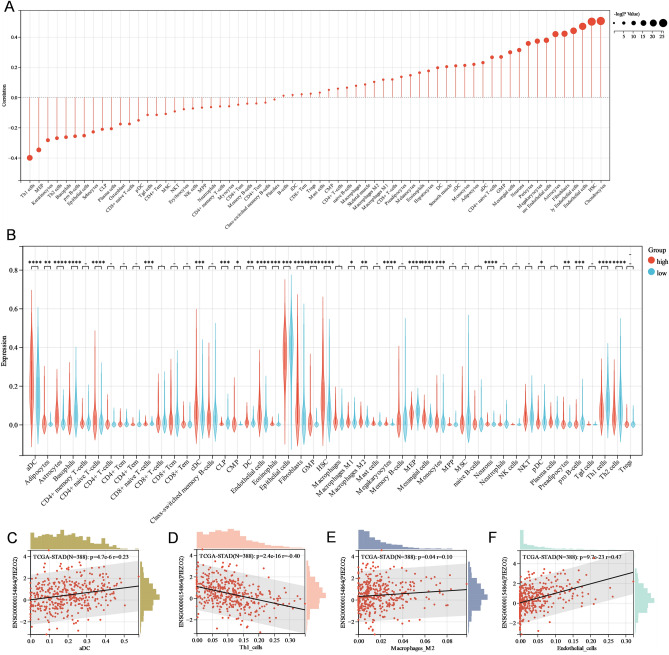
The relationship between PIEZO2 and immune cells in gastric cancer (GC) patients (**A**) Relationship between PIEZO2 and immune cell infiltration in GC. (**B**) Difference in immune cell infiltration between the PIEZO2 high and low expression groups. (**C-F**) Scatterplot showing PIEZO2 expression and correlation of infiltration degree of dendritic cells, TH1, M2 type macrophages, and endothelial cells. *p < 0.05; **p < 0.01; ***p < 0.001; ****p < 0.0001; *ns* not significant.

The degree of PIEZO2 expression and immune cell infiltration were compared. The findings revealed that PIEZO2 was positively associated with the majority of immune cell infiltration. The PIEZO2 high and low expression groups differed significantly (Fig. 4A,B), which was negatively correlated with Th1 cells, megakaryocyte-erythrocyte progenitors, keratinocytes, Th2 cells, basophils, pro-B-cells, epithelial cells, sebocytes, common lymphoid progenitors, plasma cells, osteoblasts, CD8^+^ naive T cells, plasmacytoid dendritic cells, gamma delta T cells, CD4^+^ effector memory T cells, and mesenchymal stem cells. The association between PIEZO2 and immune cell enrichment was investigated using Spearman correlation (Fig. 4C–F). It was shown to be favourably connected with M2-type macrophage infiltration and negatively correlated with endothelial cell and Th1 cell infiltration.

### The relationship between PIEZO2 and immune checkpoints in patients with GC

**Figure 5:**
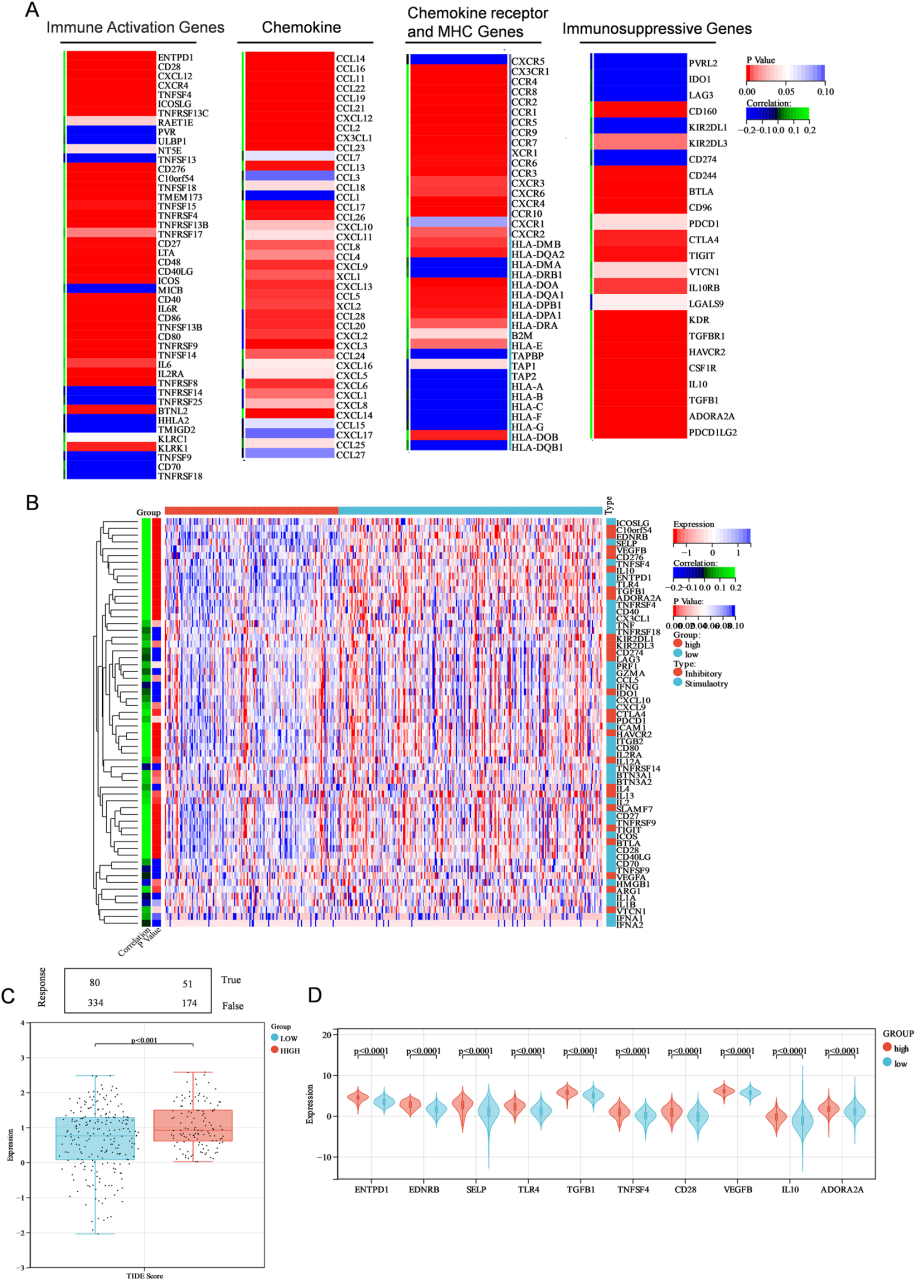
Relationship between PIEZO2 and immune checkpoint in patients with gastric cancer (**A**) Relationship between PIEZO2 and major histocompatibility complex genes, immune-activating genes, immune-suppressing genes, chemokines, and their receptors. (**B**) Differences in the expression of genes related to immune checkpoint between the PIEZO2 high and low expression groups. (**C**) Predictive response of different PIEZO2 expression groups to blocking treatment of immune checkpoint. (**D**) Correlation difference between PIEZO2 high and low expression groups. Expression level of genes related to the top 10 immune checkpoints. *p < 0.05; **p < 0.01; ***p < 0.001; ****p < 0.0001; *ns* not significant.

We also looked at the link between PIEZO2 and the expression of immune-related genes in the GC microenvironment, including MHC, immune-activating and immunosuppressive genes, chemokines, and their receptors. The findings revealed that PIEZO2 was positively linked with the expression of immune-related genes, indicating that these genes may play a role in the control of immune cell infiltration (Fig. 5A). The expression of immunological checkpoint-related genes was then compared in various PIEZO2 expression groups. ENTPD1, EDNRB, SELP, TLR4, TGFB1, TNFSF4, CD28, VEGFB, IL10, and ADORA2A were found to be considerably up-regulated in the PIEZO2 overexpression groups (Fig. 5B), and PIEZO2 was shown to be positively linked with the expression of immunological checkpoint-related genes (Fig. 5D).

### PIEZO2 might be used as an immunotherapy target in GC

To explore the value of PIEZO2 in immunotherapy. We discovered that the anticipated response rate of patients with GC in the PIEZO2 overexpression group was much lower when we used the TIDE algorithm to examine the clinical response of immune checkpoint blocking (Fig. 5C). These findings suggest that PIEZO2 might be used as an immunotherapy target.

### Functional analysis of PIEZO2 in GC

**Figure 6:**
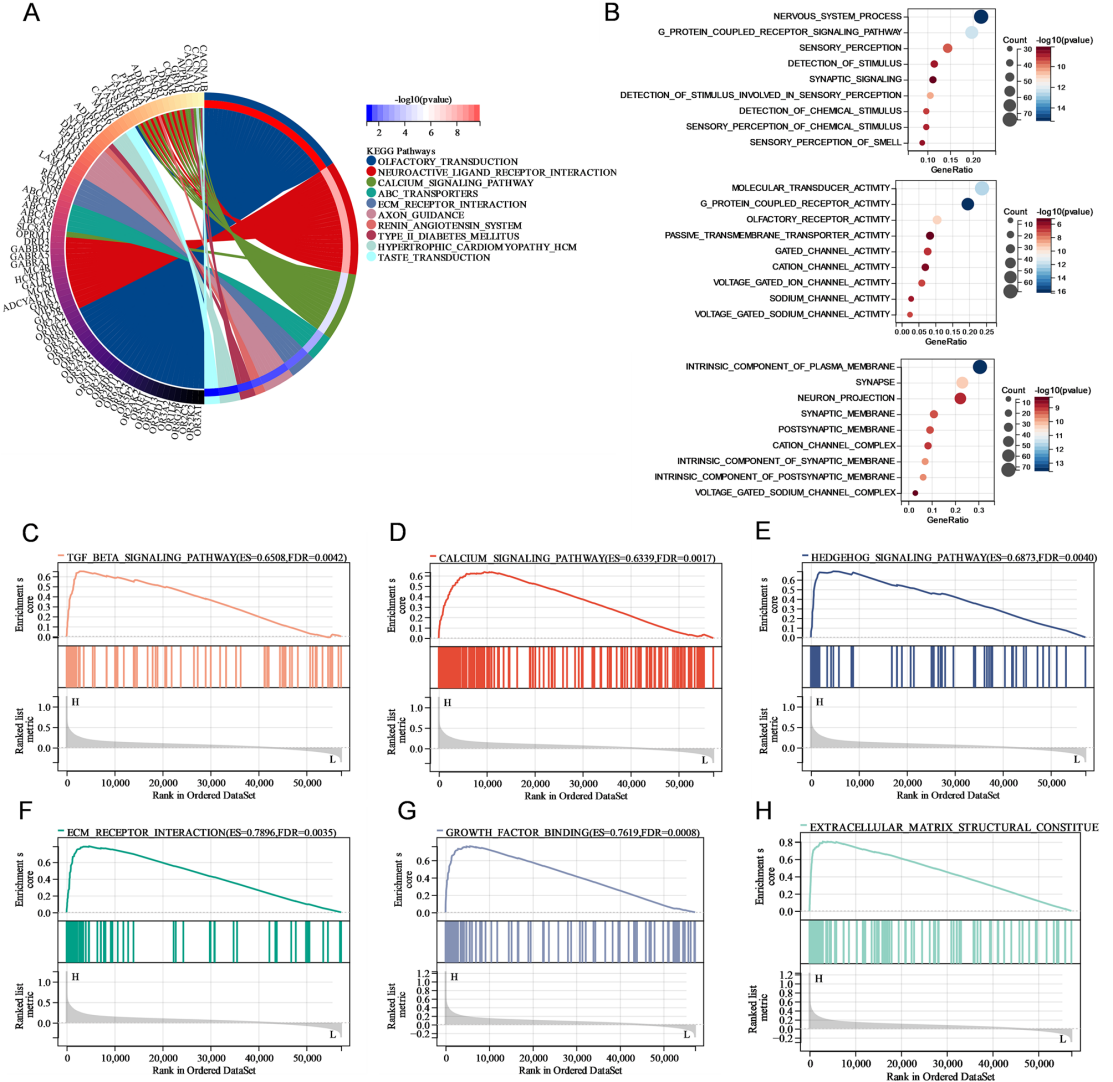
Gene Ontology (GO) classification and pathway analysis of PIEZO2 in gastric cancer (**A**) Kyoto Encyclopedia of Genes and Genomes pathway enrichment analysis identified the first 10 significant enrichment pathways. (**B**) GO annotations of differentially expressed genes (DEGs). (**C**–**H**) DEG Gene Set Enrichment Analysis used to identify PIEZO2 related signalling pathways and biological processes, including the TGF-β, calcium, hedgehog, and extracellular signalling pathways, extracellular matrix receptor interaction and growth factor binding. *p < 0.05; **p < 0.01; ***p < 0.001; ****p < 0.0001; *ns* not significant.

We performed GO classification and KEGG pathway enrichment analysis of PIEZO2-related differential genes in TCGA to further understand the probable function of PIEZO2 in GC. The findings revealed that PIEZO2-related differential genes were mostly concentrated in factory transportation, neutral light receiver interaction, the calcium signaling pathway, ATP-binding cassette transporters, and extracellular matrix (ECM) receiver interaction (Fig. 6A). In biological processes, PIEZO2-related differential genes are mainly enriched in the nervous system processes, G protein-coupled receiver signaling pathways, sensory perception, detection of stimulus, and synchronous signaling. In terms of molecular function, the PIEZO2-related differential genes are mainly related to the motor transport activity, G protein-coupled receiver activity, factory receiver activity, passive transport transporter activity, and gateway channel activity (Fig. 6B).

The PIEZO2-related signal pathway in GC was identified using GSEA. The path was shown to be strongly linked to tumor growth and metastasis based on the enrichment data. Finally, PIEZO2 is an immune-related gene that may participate in the TGF-, calcium, hedgehog, and extracellular signaling pathways, as well as ECM receptor interaction and growth factor binding, to enhance the incidence and development of GC (Fig. 6C–H).

## Discussion

The evolutionarily conserved Piezo protein composed of PIEZO1 and PIEZO2 is a large ion channel protein. PIEZO1, as a mechanical receptor, participates in the differentiation of neural stem cells and mesenchymal stem cells by sensing traction. In contrast, PIEZO2 is mainly expressed in primary sensory neurons and is related to tenderness, gentle touch, airway extension, proprioception, and heart rate regulation^18^. In previous studies, PIEZO1 was overexpressed in GC cell lines compared to normal gastric tissue. In addition, GC patients with high expression of PIEZO1 have a poor prognosis^19^. However, the relationship between PIEZO2 and poor prognosis, and immune infiltration level of patients with GC has not yet been reported. This study confirmed that PIEZO2 has a role in the degree of immune infiltration. Overexpression of PIEZO2 was associated with T stage (T2 vs T1, P < 0.001; T3, T4 vs T2, P < 0.001), N stage (N2, N3, N4 vs N1, P < 0.05), pathological stage (I vs III vs II, P < 0.05), histological grade (G1 vs G2, P < 0.01) (Supplement Fig. S1, Supplement Table S1). However, in patients with poorly differentiated GC, the high expression of PIEZO2 is related to a better prognosis. This might be because of the reduced tumor stemness and gene mutation degree in individuals with GC who express high levels of PIEZO2. According to research, PIEZO2, which is highly expressed in many tumors, can enhance tumor progression^5, 20, 21^. Interestingly, it has no statistical significance in the prognosis of females, but it is negatively correlated with the prognosis of males.

A nomogram of PIEZO2 and other clinical variables (age, gender, and clinical stage) was produced using multivariate Cox analysis. By combining recognized risk indicators, this scoring technique aims to give a more accurate prognostic evaluation for patients with GC. The calibration curve and C index reveal that the projected and actual values of the 1, 3, and 5-year total survival rates are quite consistent. As a result, the nomograph model we developed has the potential to be a useful tool for the personalized assessment of GC patients' survival.

We studied the association between immune cell infiltration and PIEZO2 expression further. The findings revealed that PIEZO2 overexpression was associated with M2 macrophages and endothelial cells. The poor prognosis of the PIEZO2 overexpression group might be attributed to an imbalance in immune function and internal environment, resulting in a loss in anti-tumor potential. The link between PIEZO2 and immune-related genes was examined to better understand the probable mechanism associated with PIEZO2 and immune cell infiltration. The findings revealed that the expression of the majority of immune-related genes was positively linked with PIEZO2. As a result, we may conclude that the poor prognosis of individuals with high PIEZO2 expression is associated with the overexpression of immunosuppressive genes. TIDE score and expression of CD274, CTLA4, HAVCR2, LAG3, PDCD1, PDCD1LG2, and TIGIT were greater in GC patients with up-regulated PIEZO2 expression than in individuals with low PIEZO2 expression. A previous study showed that higher TIDE scores in patients indicated a T cell microenvironment in dysfunctional tumors, which is not only related to the poor blocking treatment of immune checkpoints but also related to the lower survival rate under anti-PD-1 and anti-CTLA4 treatment^22^. The findings of these studies suggest that targeting PIEZO2 in the clinical treatment of GC patients may be a viable technique for inhibiting the treatment of immunological checkpoints.

We selected DEGs linked to PIEZO2 and performed GO function annotation, GSEA, and KEGG pathway enrichment analysis to investigate the biological role of PIEZO2 in GC. The GSEA suggested that the overexpression of PIEZO2 in the calcium, Hedgehog, and TGF- β signaling pathways, ECM receptor, the activity of cell adhesion medium, calcium ion and cytokine binding, and structural composition of the extracellular mechanism are closely related. Based on the results of enrichment analysis, we can infer that PIEZO2 promotes the development of GC through such faulty processes as immune response, regulation of cell–matrix, and inflammatory response. PIEZO2 promotes the development of GC through fault processes such as immune response, regulation of cell–matrix, and inflammatory response. This provides a direction for further research on the mechanism of PIEZO2 in GC.

This study revealed the role of PIEZO2 in immune cell infiltration and immune-related gene expression in GC. However, there are certain limitations to this study, as all samples are based on RNA sequencing data from internet sources. Data from several systems are diverse. Some subgroups in subgroup analysis have an insufficient sample size, therefore more samples are required for verification. The role of PIEZO2 in the prognosis of male and female patients has to be investigated further. The link between PIEZO2 expression and immune cell infiltration and immune-related gene expression has to be investigated further to ensure the veracity of the findings.

This study found that PIEZO2 is associated with the poor prognosis of GC patients, which may be caused by the imbalance of the immune function internal environment caused by PIEZO2. PIEZO2 was identified as a potential new biomarker for the diagnosis and prognosis of patients with GC. In decision-making about diagnosis and treatment with GC patients, the expression of PIEZO2 in GC can predict the histological grading and treatment effectiveness of blocking treatment of immune checkpoints. The established survival model can be used as a practical tool to personalize the prognosis of patients with GC. Through enrichment analysis, it was found that PIEZO2 promotes the development of GC through fault processes such as immune response, regulation of cell–matrix, and inflammatory response, so targeting PIEZO2 may be a potential target for GC therapy. This work provides a new and feasible direction for clinical diagnosis, prognosis prediction, and immunotherapy of GC patients. Further experiments can be conducted to explore the specific mechanism of PIEZO2 in GC.

### Supplementary Information

Supplementary Information.

## Data Availability

The datasets analyzed during this study are available in the TCGA repository (https://portal.gdc.cancer.gov/) and the GEO database (https://www.ncbi.nlm.nih.gov/geo/query/acc.cgi?acc=gse54129).
