# Supplementary material for: PIEZO2 expression is an independent biomarker prognostic for gastric cancer and represents a potential therapeutic target
Source: Sci Rep. 2024 Jan 12;14:1206. doi: 10.1038/s41598-023-48577-5 (PMC10786914; doi:10.1038/s41598-023-48577-5)
Supplement: Supplementary file 1 — Supplementary Information. [file 41598_2023_48577_MOESM1_ESM.docx]

**Supplement Figure**


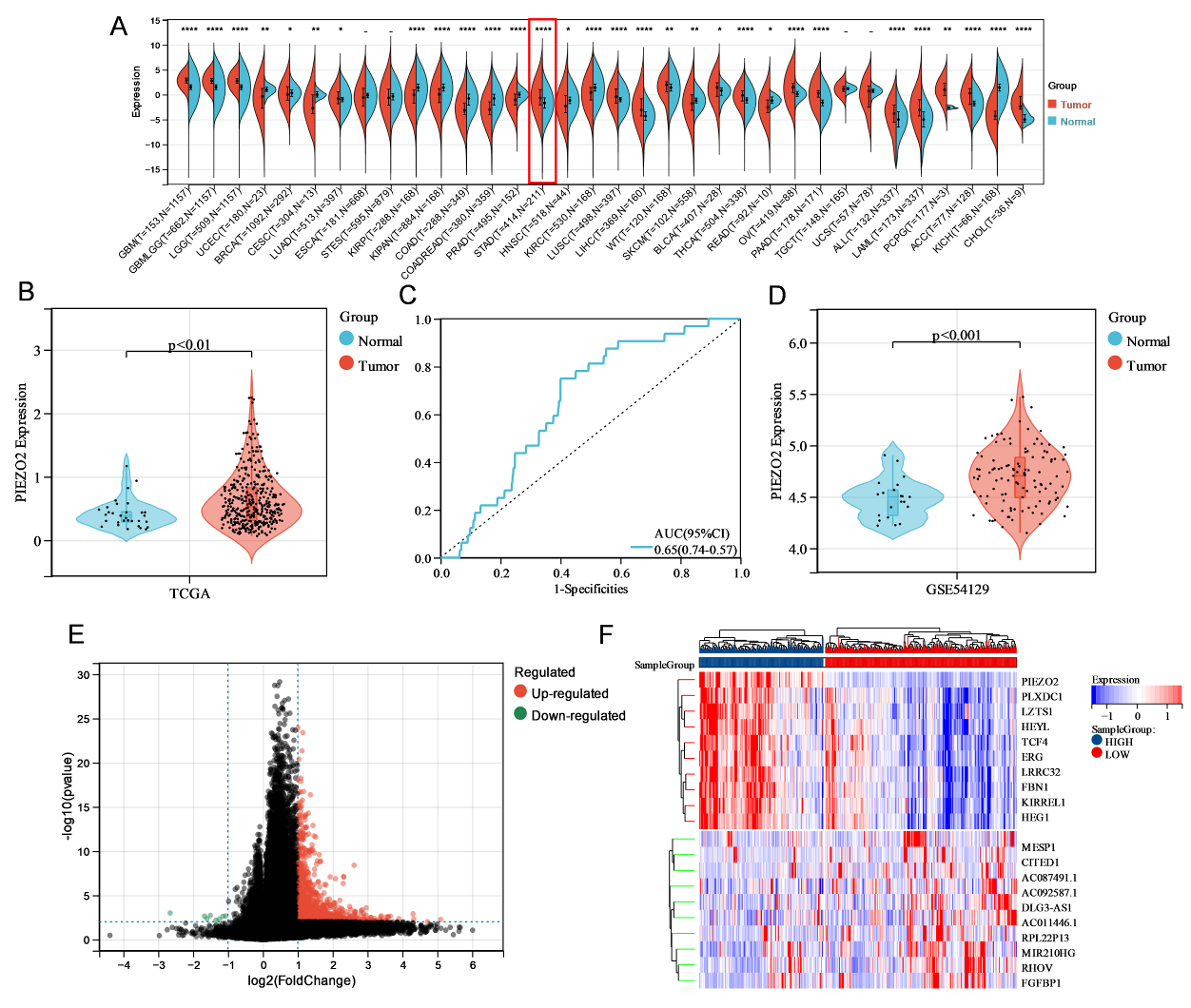


Supplement Fig. 1. PIEZO2 is overexpressed in gastric cancer (GC) tissues (A) Pan-cancer analysis of PIEZO2 expression in tumor and normal tissues in The Cancer Genome Atlas (TCGA) and Genotype-Tissue Expression databases, the expression of PIEZO2 in tumor tissues of patients with GC is higher than that of normal patients. (B) Expression level of PIEZO2 between GC tissues and adjacent normal tissues in TCGA, the expression of PIEZO2 in tumor tissues of patients with GC is higher than that of normal patients. (C) Receiver operating characteristic curve predicts the sensitivity and specificity of PIEZO2 to distinguish between GC tissues and normal tissues, showing that PIEZO2 has a certain value in the prediction of GC. (D) Expression level of PIEZO2 between GC tissues and adjacent normal tissues in GSE54129, the expression of PIEZO2 in tumor tissues of patients with GC is higher than that of normal patients. (E) Volcanic map showing PIEZO2 related differentially expressed genes (DEG) based on TCGA database. Red dots and green dots represent up and down-regulated genes, respectively. (F) Heat map showing the first 20 DEGs related to PIEZO2. * p < 0.05; ** p < 0.01; *** p < 0.001; **** p < 0.0001; ns, not significant. ACC: adrenocortical carcinoma; BLCA: bladder urothelial carcinoma; BRCA: breast invasive carcinoma; CESC: cervical squamous cell carcinoma and endocervical adenocarcinoma; CHOL: cholangiocarcinoma; COAD: colon adenocarcinoma; DLBC: lymphoid neoplasm diffuse large B-cell lymphoma; ESCA: oesophageal carcinoma; GBM: glioblastoma multiforme; HNSC: head and neck squamous cell carcinoma; KICH: kidney chromophobe; KIRC: kidney renal clear cell carcinoma; KIRP: kidney renal papillary cell carcinoma; LAML: acute myeloid leukaemia; LGG: brain lower grade glioma; LIHC: liver hepatocellular carcinoma; LUAD: lung adenocarcinoma; LUSC: lung squamous cell carcinoma; MESO: mesothelioma; OV: ovarian serous cystadenocarcinoma; PAAD: pancreatic adenocarcinoma; PCPG: pheochromocytoma and paraganglioma; PRAD: prostate adenocarcinoma; READ: rectum adenocarcinoma; SARC: sarcoma; SKCM: skin cutaneous melanoma; STAD: stomach adenocarcinoma; TGCT: testicular germ cell tumors; THCA: thyroid carcinoma; THYM: thymoma; UCEC: uterine corpus endometrial carcinoma; UCS: uterine carcinosarcoma; UVM: uveal melanoma.


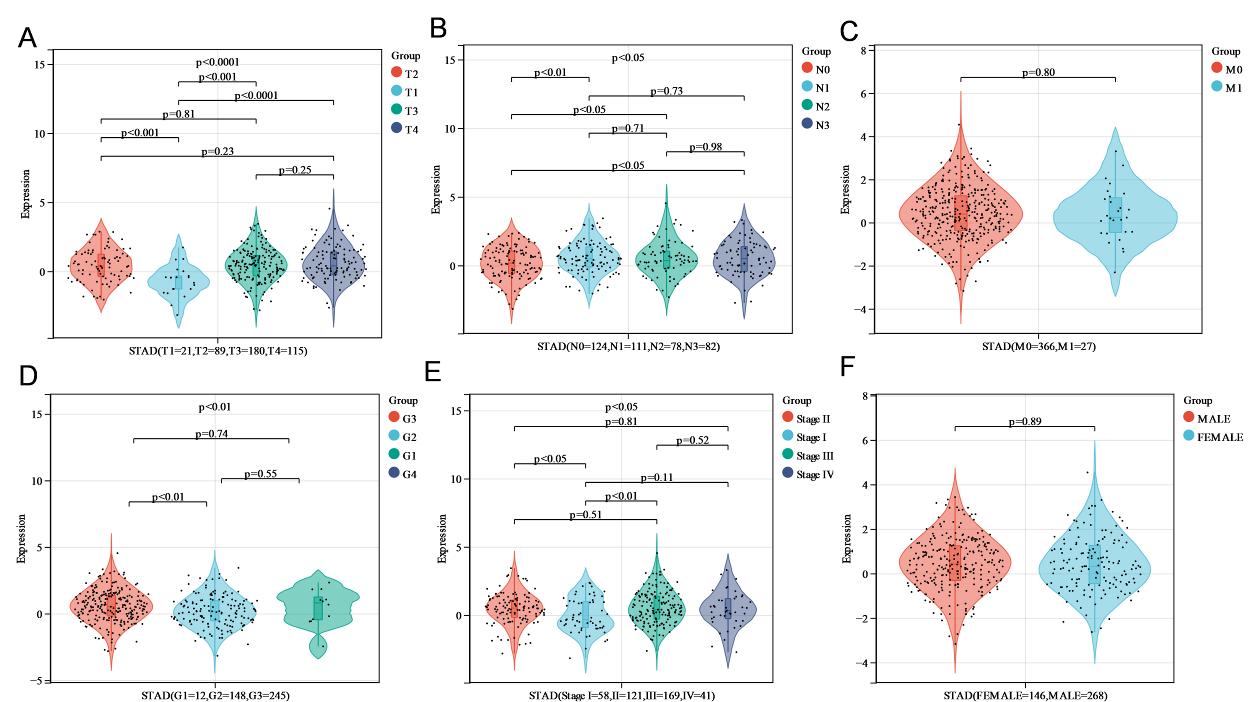


Supplement Fig. 2. Relationship between PIEZO2 expression level and clinical pathological characteristics in patients with gastric cancer (GC) (A) Overexpression of PIEZO2 is associated with the T stage of GC (T2 and T1, *P<*0.001; T3, T4 and T2, *P<*0.001). (B) Overexpression of PIEZO2 is associated with the N stage of GC (N2, N3, N4 and N1, *P<*0.05). (C) M stage. (D) PIEZO2 overexpression and clinical stage of GC (G1 vs. G2, *P<*0.01) (E) PIEZO2 overexpression and GC histological grade (Phase I and III vs phase II, *P<*0.05); (F) Overexpression of PIEZO2 has no significant relationship with the sex of patients with gastric cancer (P>0.05). * p < 0.05; ** p < 0.01; *** p < 0.001; **** p < 0.0001; ns, not significant.


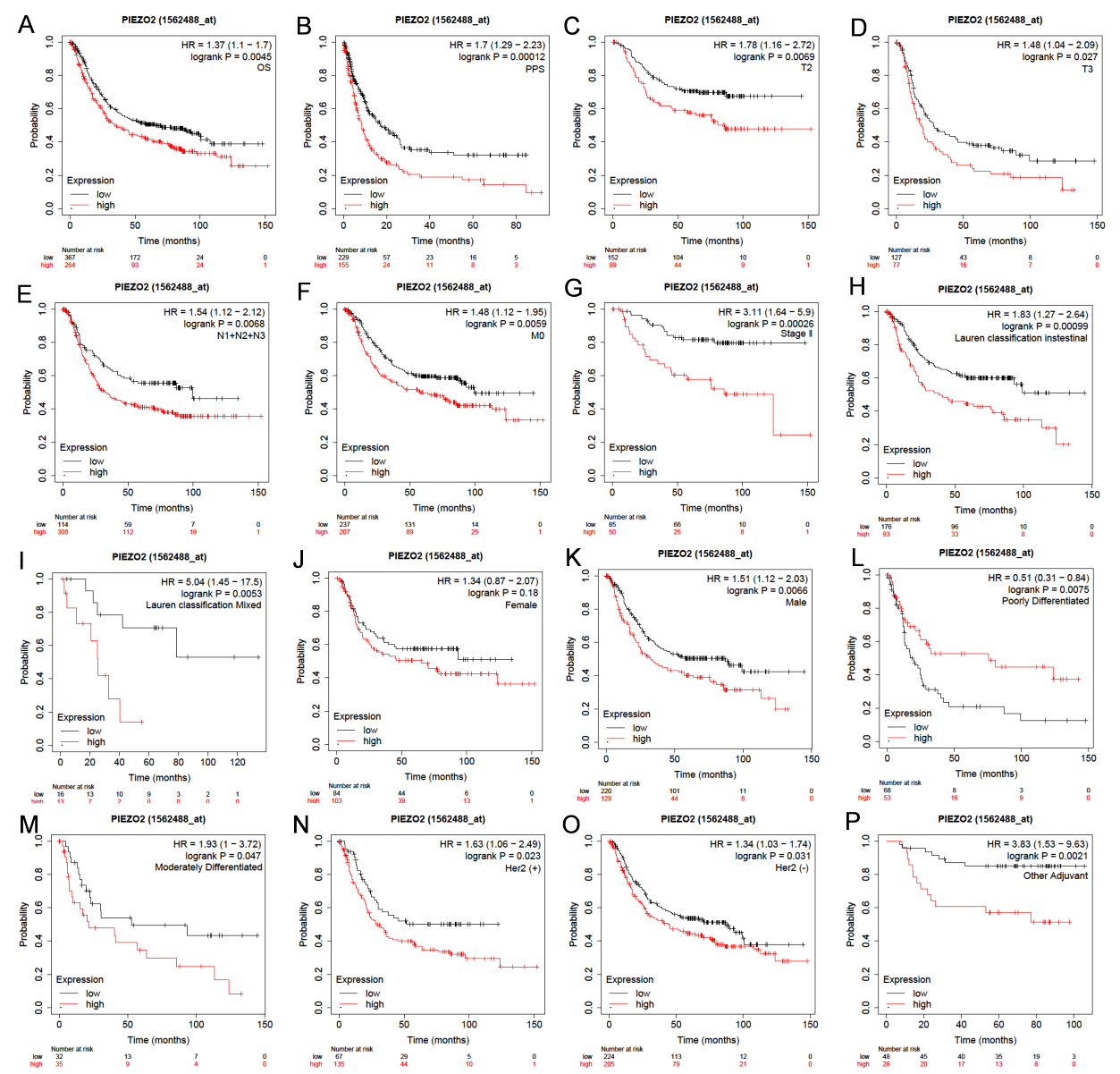


Fig. 3. Kaplan–Meier analysis of patients with gastric cancer according to GGT5 expression level and subgroup analysis. (A) Negative relationship between PIEZO2 expression and overall survival. (B) Negative relationship between PIEZO2 expression and post-progression survival. (C-P) Negative relationship between PIEZO2 expression and patient. (D-P) Comparison of total survival curve of T2, T3, N1+N2+N3, M0, Stage I, Lauren classification experimental, Lauren classification mixed, poorly differentiated, moderately differentiated, female, male, Her2 (+), Her2 (-), and treatment, and other adaptive subgroups between PIEZO2 high and low expression groups, which positively correlates with poorly differentiated tumors in patients with GC.

# Supplement Tables

| **Table1 Differential Analysis of Gene Expression** | | | |
| --- | --- | --- | --- |
|  | **Tumor** | **Normal** | **P value** |
| **Up Regulation:** | | | |
| LGG | 0.70±1.57 | 0.44±1.88 | 1.30E-03 |
| BRCA | 1.33±1.89 | 0.75±1.54 | 2.30E-05 |
| STES | 0.38±1.25 | -0.21±1.43 | 1.30E-15 |
| KIPAN | 0.95±2.20 | -0.12±1.39 | 4.10E-11 |
| STAD | 0.44±1.20 | -0.92±1.38 | 2.50E-33 |
| HNSC | -1.05±1.74 | -2.39±1.38 | 2.00E-07 |
| KIRC | 2.19±1.45 | -0.12±1.39 | 1.20E-51 |
| LIHC | 1.64±1.54 | 0.46±0.98 | 8.90E-21 |
| WT | 1.85±1.16 | -0.12±1.39 | 1.60E-28 |
| PAAD | 1.07±1.36 | -0.29±1.88 | 3.00E-15 |
| ALL | -5.33±3.13 | -7.98±2.52 | 1.50E-17 |
| LAML | -0.52±3.60 | -7.98±2.52 | 1.60E-63 |
| PCPG | 0.96±1.77 | -1.38±0.92 | 0.02 |
| CHOL | 2.13±1.94 | 0.73±0.42 | 0.01 |
| **Down Regulation:** | | | |
| GBM | 0.10±1.41 | 0.44±1.88 | 0.02 |
| UCEC | -3.07±2.08 | -0.51±1.44 | 5.90E-09 |
| CESC | -2.90±1.91 | -0.23±1.15 | 1.40E-06 |
| LUAD | 0.70±1.38 | 2.87±1.23 | 7.90E-97 |
| KIRP | -1.04±1.84 | -0.12±1.39 | 9.50E-13 |
| COAD | -1.19±1.53 | 1.17±2.15 | 1.20E-47 |
| COADREAD | -1.05±1.52 | 1.15±2.13 | 8.60E-51 |
| PRAD | 0.05±1.30 | 1.75±1.17 | 2.20E-37 |
| LUSC | -0.09±1.57 | 2.87±1.23 | 5.30E-115 |
| BLCA | -1.16±1.78 | 0.92±1.26 | 3.00E-09 |
| READ | -0.61±1.42 | 0.35±0.98 | 0.02 |
| OV | -1.33±1.91 | -0.24±1.41 | 1.50E-07 |
| TGCT | 0.32±1.55 | 1.09±1.06 | 3.00E-09 |
| KICH | -0.35±1.25 | -0.12±1.39 | 0.05 |

| **Table2 Results of Kaplan-Meier Analysis** | | | |
| --- | --- | --- | --- |
|  | **HR** | **CI** | **P Value** |
| **OS** | 1.37 | 1.1-1.7 | 0.0045 |
| **PPS** | 1.7 | 1.29-2.23 | 0.00012 |
| **T2** | 1.78 | 1.16-2.27 | 0.0069 |
| **T3** | 1.48 | 1.04-2.09 | 0.024 |
| **N1+N2+N3** | 1.54 | 1.12-2.12 | 0.0068 |
| **M0** | 1.48 | 1.12-1.95 | 0.0059 |
| **Stage ‖** | 3.11 | 1.64-5.9 | 0.00026 |
| **Lauren classification  instestinal** | 1.83 | 1.27-2.64 | 0.00099 |
| **Lauren classification mixed** | 5.04 | 1.45-17.5 | 0.0053 |
| **Poorly  Differentiated** | 0.51 | 0.31-0.84 | 0.0075 |
| **Moderately  Differentiated** | 1.93 | 1-3.72 | 0.047 |
| **Female** | 1.34 | 0.87-2.07 | 0.18 |
| **Male** | 1.51 | 1.12-2.03 | 0.0066 |
| **Her2 (+)** | 1.63 | 1.06-2.49 | 0.023 |
| **Her2 (-)** | 1.34 | 1.03-1.74 | 0.031 |
| **Treatment:  other adjuvant** | 3.83 | 1.53-9.63 | 0.0021 |
